# Bilateral Vocal Cord Carcinoma in a Sarcoidosis Patient during Infliximab Therapy

**DOI:** 10.1155/2013/308092

**Published:** 2013-05-15

**Authors:** Adriane D. M. Vorselaars, Elisabeth V. Sjögren, Coline H. M. van Moorsel, Jan C. Grutters

**Affiliations:** ^1^Centre of Interstitial Lung Diseases, Department of Pulmonology, St. Antonius Hospital, Koekoekslaan 1, 3435 CM Nieuwegein, The Netherlands; ^2^Department of Otolaryngology, Head & Neck Surgery, Leiden University Medical Centre, Albinusdreef 2, 2333 ZA Leiden, The Netherlands; ^3^Division of Heart and Lungs, University Medical Centre Utrecht, Heidelberglaan 100, 3584 CX Utrecht, The Netherlands

## Abstract

*Introduction*. Although the role of TNF-**α** in tumor development is not fully understood, an increased risk of malignancy with TNF-**α**-inhibitors, such as infliximab, has been suggested. *Case Presentation*. We present a 54-year-old nonsmoking female sarcoidosis patient. After seven months of infliximab therapy a T1aN0M0 larynx carcinoma of the right vocal cord was found and excised. Within a year, whilst still on treatment, a second larynx carcinoma of the opposite vocal cord appeared. *Discussion*. A bilateral vocal cord tumor is rare, especially in a never smoker. Evidence on the role of infliximab in carcinogenesis is inconclusive. To date, there are no follow-up studies evaluating malignancy risk of infliximab therapy in sarcoidosis patients. No studies in other diseases focus on laryngeal carcinomas during infliximab use. We argue that infliximab treatment might have attributed to the rapid progression of vocal cord carcinomas in this patient with an a priori low risk tumor profile. This case illustrates that caution remains warranted in patients with previous malignancies when considering initiation of TNF-**α**-inhibitors.

## 1. Introduction

Tumor necrosis factor-*α* (TNF-*α*) inhibitors such as infliximab are used in treatment of various diseases like rheumatoid arthritis, Crohn's disease, ankylosing spondylitis, and psoriasis. Today, infliximab is also an upcoming therapeutic option for cases of severe pulmonary and/or extrapulmonary sarcoidosis refractory to standard therapy [1].

Besides its key role in inflammation, TNF-*α* has several qualities that may have impact on carcinogenesis, tumor growth, and the time point of clinical detection of malignancies. Although the role of TNF-*α* in tumor development is not fully understood, an increased risk of malignancy with TNF-*α*-inhibitors has been suggested in other diseases than sarcoidosis [2–4]. 

## 2. Case Report

We present the case of a 54-year-old female who was diagnosed five years earlier with Scadding stage II sarcoidosis. Diagnosis was biopsy proven with noncaseating granulomas found in endobronchial biopsies. Pulmonary complaints were dyspnoea on exertion and coughing. Moreover, her sarcoidosis was accompanied by severe small fiber neuropathy which was invalidating in everyday life. Other extrapulmonary symptoms were extrathoracic lymph nodes. A trial of prednisone failed due to severe psychological side effects. The severity of her symptoms and established disease activity on ^18^F-fluorodeoxyglucose positron emission tomography (^18^F-FDG-PET) scan prompted the decision to initiate infliximab therapy. Infliximab was administered at a monthly dose of 5 mg/kg bodyweight accompanied by a low dose of methotrexate (7.5 mg/week).

She visited an Ear-Nose-Throat (ENT) specialist for complaints of varying hoarseness. She had never smoked and did not consume alcohol. There was no history of passive smoking or occupational exposure as she had an office job. Biopsy of the vocal cord did not reveal an underlying malignancy and it was diagnosed as chronic laryngitis. She was treated with speech therapy, proton pump inhibitors, and antifungal therapy. 

**Figure 1:**
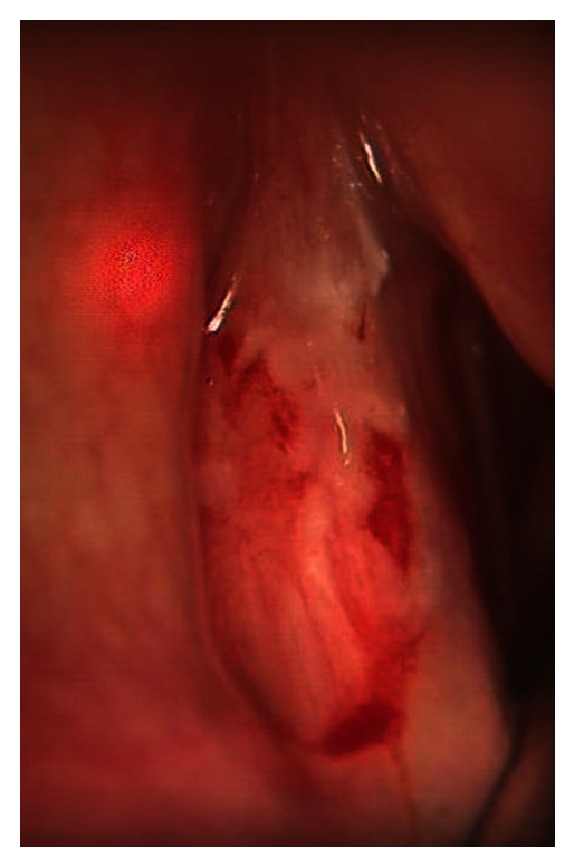
Left sided T1aN0M0 vocal cord carcinoma under surgical microscope.

**Figure 2:**
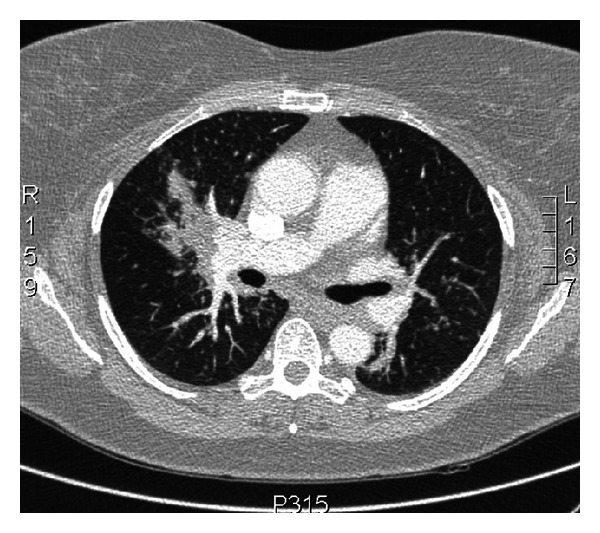
HRCT of the thorax after discontinuation of infliximab showing increased consolidation and nodular abnormalities.

**Figure 3:**
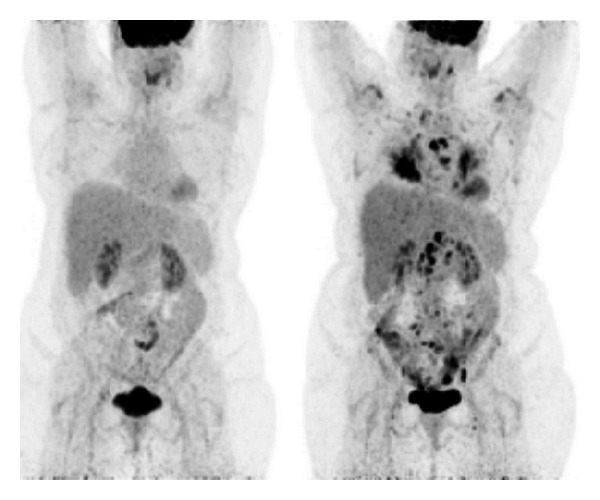
^18^F-FDG-PET scan monitoring sarcoidosis activity level. In the left frame is shown the ^18^F-FDG-PET scan during treatment with infliximab and in the right frame the ^18^F-FDG-PET scan after discontinuation of infliximab revealing reactivation of sarcoidosis as black spots in lungs and lymph nodes.

Seven months after initiation of infliximab therapy she revisited her ENT specialist with increasing vocal problems. Biopsy of the right true vocal cord revealed a squamous cell carcinoma with extensive necrosis, which was radically excised using laser surgery. After staging of the lymph nodes and exclusion of pulmonary metastases by chest X-ray, it was classified as a T1aN0M0 larynx carcinoma of the right true vocal cord. After elaborate discussions, she continued infliximab therapy because of the unlikely relation with the vocal cord tumor, full recovery from her vocal cord malignancy after ENT surgery, and the positive effect on her sarcoidosis with increased quality of life. Within a year her voice turned increasingly hoarse and a second larynx carcinoma on the opposite vocal cord was discovered (Figure 1). This vocal cord tumour was treated with laser excision as well. Infliximab therapy was now discontinued on behalf of the uncertain but possible relationship between infliximab and the bilateral larynx carcinomas. At the moment of the second ENT surgery her sarcoidosis symptoms were stable. Unfortunately, half a year after infliximab discontinuation the small fiber neuropathy and pulmonary symptoms returned as shown on High Resolution CT of the chest (Figure 2) and on ^18^-FDG-PET scan which revealed extensive systemic reactivation of sarcoidosis (Figure 3).

## 3. Discussion

Laryngeal cancer is generally uncommon in males and very rare in females being the 22nd most frequent cancer in females. Vocal fold cancer constitutes half of laryngeal cancers. The highest rate in females is found in the United States African American population and is 3/100.000 [5]. Vocal fold squamous cell carcinomas most often develop on healthy mucosa, but precancerous lesions can occur. The main risk factor is smoking of tobacco; alcohol consumption further increases the risk especially when combined with smoking [5].

A bilateral vocal cord carcinoma is extremely rare, especially in a patient without risk factors such as smoking or alcohol use. In patients with a larynx carcinoma, the rate of development of a second metachronous primary cancer of the upper aerodigestive tract is around 1% per year. Patients with a history of smoking by far had the highest risk in this group [6].

To date, there are no follow-up studies evaluating malignancy risk of infliximab therapy in sarcoidosis patients. No studies in other diseases focus on laryngeal carcinomas during infliximab use. Studies on the possible increased risk of malignancy by infliximab have been performed in other diseases with varying outcomes. Some studies and case reports did suggest a relationship between infliximab and malignancy [2–4]. Several larger studies performed in Crohn's disease, rheumatoid arthritis, and psoriasis did not find an increased risk [7–9]. One large meta-analysis including 22.904 patients treated with different TNF-*α*-inhibitors only found an increased risk of nonmelanoma skin cancer but no increased risk of other cancers [10].

Because the association between infliximab and malignancy has not been elucidated, we did not stop treatment with infliximab after the first vocal cord carcinoma occurred. This is in line with the Dutch guideline on responsible use of biologicals which does not determine whether previous malignancy is a strict contraindication. Moreover she fully recovered after the first ENT surgery and infliximab was the only therapy that significantly improved the patients symptoms of sarcoidosis. However, in retrospect this might not have been the right decision for this patient. 

It could be possible that the carcinogenic effect of infliximab only affects certain patients with an underlying genetic susceptibility. We argue that infliximab treatment might have contributed to the rapid development of vocal cord carcinomas in this patient with an a priori low risk tumor profile, but currently unknown genetic susceptibility. In this apparently malignancy prone patient we therefore did not restart infliximab therapy for the relapse of her sarcoidosis symptoms. 

This case illustrates that even though guidelines do not provide strict contra-indication, caution is warranted in patients developing their first malignancy shortly after initiation of infliximab therapy and in patients with previous malignancies when considering initiation of TNF-*α*-inhibitors. More knowledge should be gained on tumor risk after infliximab therapy in patients with previous malignancies, possibly discouraging use of TNF-*α*-inhibitors in future cases.

## Abbreviations

TNF-*α*: Tumor necrosis factor-*α*
ENT: Ear Nose Throat
^18^F-FDG-PET: ^18^F-fluorodeoxyglucose positron emission tomography.
